# Has Cotton Grass a Commercial Value?

**Published:** 1917-07-21

**Authors:** 


### HAS COTTON GRASS A COMMERCIAL VALUE?

The marshy moorlands of the country above the cultivation line are white with the flower of the cotton grass, moorflower, hare's-tail grass, or *Eriophorum vaginatum*. To a correspondent, as to many others before him, has occurred the idea that in this silvery fibre may lie a material of exceptional value in wartime. At the present, at any rate, with the sun shining upon it, the material looks abundant enough, even if it cannot be agreed that the supply is practically illimitable. Children could gather it, the correspondent contends, and cart-loads could be obtained from near Wareham (Dorset), and from Northumberland, Cumberland, Westmorland, Yorkshire, Wales, Scotland, and Ireland.

The material is plentiful upon the Pennines, and can be plucked freely within sight of the chimneys of Oldham, the largest cotton-spinning town in the world, and of those of Huddersfield, where there are manufacturers with an acute eye for fibre which can conceivably be used worked up with woollen. The grass blows neglected, and if failure to use it gives no proof of its inutility the neglect at least suggests that it is not a ready substitute for the well-known textile fibres, for it is not one that could readily have been overlooked. The filaments are brittle and give way under a very light pull. Their sides are sleek, and there is an absence of the natural twist which gives one cotton fibre a purchase upon its neighbours and allows of the production of a coherent spun thread. The fibre is not naturally designed for spinning and weaving, but it is suggested that it might have a value for surgical dressings.

Absorbency is one of the most elementary requisites in surgical dressings, and in a state of nature at least the cotton grass is lacking in this quality. Thrown upon the surface of water the filaments show an invincible disposition to float, whereas part of the character of a good dressing is that it should take up water freely, become waterlogged quickly, and eventually sink. Comparative tests made against the recognised dressings tell anything but in favour of this down, and suggest even that its true sphere may be as an unsinkable rival to kapok for the filling of lifesaving cushions and waistcoats. Cotton only becomes absorbent by being bleached and freed from its waxy external coating, and it is conceivable that under a parallel treatment the affinity of the grass flower for water might be improved. Even so, the serviceability of the material as a dressing is in economic doubt. Cotton grass could be gathered, but so could wood to make wood wool, or so could soft cotton rags. Again, the still more abundant peat moss, above which the cotton grass flowers once a season, could be collected in yet larger quantities, and perhaps to no worse purpose. Cotton grass collected and delivered at a railway station would possibly be less inexpensive than one is tempted to think. The flower is prominent just now, and especially to the eye at ground level, but there are spaces in between the heads, and no great weight of fibre is carried on each. One ton of the fibre would certainly take more collection than a ton of material of more use when obtained. The idea that the flower will make somebody's fortune is not less than thirty years old, but the fortune goes unearned.

